# Transformational Teaching in Physical Education and Students’ Leisure-Time Physical Activity: The Mediating Role of Learning Climate, Passion and Self-Determined Motivation

**DOI:** 10.3390/ijerph17134844

**Published:** 2020-07-05

**Authors:** Isabel Castillo, Javier Molina-García, Isaac Estevan, Ana Queralt, Octavio Álvarez

**Affiliations:** 1 Department of Social Psychology, University of Valencia, 46010 Valencia, Spain; isabel.castillo@uv.es; 2 AFIPS Research Group, University of Valencia, 46022 Valencia, Spain; javier.molina@uv.es (J.M.-G.); isaac.estevan@uv.es (I.E.); ana.queralt@uv.es (A.Q.); 3 Department of Teaching of Musical, Visual and Corporal Expression, University of Valencia, 46022 Valencia, Spain; 4 Department of Nursing, University of Valencia, 46010 Valencia, Spain

**Keywords:** transformational teaching, motivation, passion, physical activity, adolescence

## Abstract

In the context of education, this study examined the relationship between perceiving a transformational physical education (PE) teacher and student’s leisure-time physical activity (PA). Furthermore, we tested the potential mediation role of motivational learning climate, passion and self-determined motivation in this relationship. The sample was composed of 2210 high-school PE students (1145 males, 1065 females) between 16 and 20 years of age. Results of structural equation modeling revealed that the perceived transformational PE teacher–PA outcomes relationship was stronger when students perceived a task-involving climate, when they were harmoniously passionate, and when they were self-determined. We conclude that students’ health-enhancing behaviours could be improved if their PE teachers use transformational teaching style and created a task-oriented learning climate.

## 1. Introduction

Adolescence is a critical period in which to consolidate healthy and physically active lifestyles that can be maintained in adulthood [1]. The positive effect of physical activity (PA) on public health is well-documented [2,3], especially in high-risk children and adolescents [4]. Research findings [5,6] indicate high levels of physical inactivity among adolescents, which increases the risk of developing obesity and other non-communicable diseases [7]. Globally, 81% of adolescents between 11–17 years old were insufficiently physically active in 2016 [8]. Moreover, in the Spanish context, 29.8% of adolescents (11–18 years old) reported a PA frequency of once a week or less in their leisure time, and only 11.6% reported vigorous PA every day [9]. In addition, prospective studies have demonstrated that PA levels decrease during the transition from adolescence to early adulthood [10].

Children and adolescents spend a lot of time in school during a long period in their lives (i.e., at least 10 years). Thus, schools have been identified as relevant institutions for promoting health, well-being, and a healthy lifestyle in young students from 5–18 years old [11,12]. Health promotion is one of the main objectives of school-based intervention programs [13]. In particular, school-based physical education (PE) has been suggested to have a valuable role in public health [14,15,16] and in motivating adolescents to have active lifestyles [17]. According to authors such as Trudeau and Shephard [18], adolescents’ adoption of an active lifestyle is influenced by positive experiences in PE. Thus, PE teachers influence their adolescent students’ motivation for PE lessons through their interactions with the students and by creating positive experiences [19]. In fact, considerable empirical support has shown that positive experiences in PE classes positively influence students’ participation in PA during their leisure time (e.g., [20,21]). Therefore, policies to increase PA aim to ensure that children and adolescents receive quality PE that helps them to develop patterns of behaviour that will keep them physically active throughout their lives [11]. However, although school-based PE programs are important in promoting PA, children and adolescents are not active enough [8,9], and obesity has significantly increased. Hence, a high priority for the World Health Organization (WHO) is to reduce this prevalence and reverse the increase in obesity [22]. In a recent review, Love et al. [23] found that school-based interventions do not positively impact young people’s PA during the entire day, with no differences across genders. In other words, schools, as a potential context in which to influence health behaviours, are not having an impact on promoting PA during the rest of the day. Therefore, we need to increase our knowledge about how PE teachers can be more effective in promoting PA outside of PE classes.

The effectiveness of PE teachers in promoting PA behaviours among adolescents has been examined using the transformational teaching framework [24]. This type of teaching is useful for understanding the role of teachers in transforming students’ attitudes and beliefs about learning and, developing intrinsic motivation and personal growth [25,26,27,28]. It involves, “the demonstration of behaviours that empower and inspire students, transcend teachers’ own self-interests, and give students the confidence to achieve higher levels of functioning” [24] (p. 133). Specifically, transformational teaching is based on four teacher behaviours grounded in transformational leadership theory [29]: idealized influence, inspirational motivation, intellectual stimulation, and individualized consideration [30]. Idealized influence is related to the idea that a transformational teacher acts as a positive role model. Inspirational motivation is reflected in the teacher’s ability to inspire students to take action to create a shared vision that is aligned with the goals and needs of the group. Individual consideration is related to the teacher’s ability to understand students’ strengths and needs and satisfy their personal goals. Finally, intellectual stimulation consists of stimulating students to use multiple perspectives to approach new and old issues.

The relationship between transformational teaching in school-based PE and adolescent engagement in PA behaviours has been analysed in a conceptual model developed by Beauchamp and Morton [24]. This model suggests that transformational teaching in PE improves PA engagement in adolescents, not only during class time (proximal effect) but also during out-of-school leisure-time (distal effect). Thus, a key factor in enhancing students’ PA inside and outside the school environment is to improve our knowledge about the influence of PE teachers’ use of a transformational teaching style on adolescents’ behavioural, cognitive and affective outcomes (e.g., [24]).

Another construct that can significantly influence students’ behaviours is the perceived learning motivational climate created by PE teachers. Achievement goal theory (AGT; [31,32]) suggests that two different perceptions of the motivational climate can be created by the teacher. These perceptions consist of a task-involving climate and an ego-involving climate, with each having different impacts on students’ behavioural, cognitive, and affective outcomes. A task-involving climate is more likely to be perceived by the students if the teacher emphasizes self-referenced improvement, individual progress, learning, cooperation, effort, and persistence; whereas an ego-involving climate will probably be perceived if the teacher encourages normative comparisons between students as the criteria for success [31,33,34]. In the PE setting, a task-involving climate has been related to adaptive outcomes such as enjoyment of activities, intrinsic motivation, higher perceived competence, interest in PE, intention to be physically active, and more positive attitudes toward PE, whereas an ego climate has been associated with maladaptive outcomes such as low effort, boredom, higher anxiety, and learned helplessness (e.g., [35,36,37,38,39,40,41,42]). Therefore, it is important to identify determinants of the task climate that can facilitate positive consequences for students’ outcomes. Based on this background, Duda and Balaguer [43] offered a framework that integrates motivational climate and leadership behaviours, suggesting that there is a strong relationship between perceptions of leadership style and motivational factors. Recently, Alvarez et al. [44], in the sports context, reported that leadership behaviours can be considered determinants of motivational climate, and they confirmed that a perception of transformational leadership is a positive predictor of a task-involving climate. In the school context, Wang [45] showed the positive effect of transformational leadership on school climates (i.e., innovation, justice, and affiliation) and students’ development.

Self-determination theory (SDT; [46]) is a theoretical framework the popularity of which in the PE context has increased in the past decade [47]. SDT makes it possible to understand why people start behaviours and persist in them [47,48]. It distinguishes among three types of motivation: intrinsic, extrinsic, and amotivation. Intrinsic motivation, the highest degree of self-determined motivation, occurs in situations where students feel free to commit to activities that they find interesting and that offer them the chance to learn. In contrast, extrinsic motivation takes place when students value the results associated with the task more than the task itself. SDT considers four types of extrinsic motivation in terms of the level of self-determination inherent in each. External regulation, which represents the lowest level of self-determination (the behaviour is controlled by the teachers, rewards, coercion, etc.); introjected regulation (the behaviour is internally controlled to avoid shame or guilt); identified regulation (the student acts because the goal is personally important); and integrated regulation (the behaviour is congruent with other values and goals). Finally, amotivation refers to the absence of the intention to act; the students do not value the activity or do not feel competent to do it [48]. 

In the case of leisure-time PA, the Beauchamp and Morton [24] conceptual model indicates the existence of mediating mechanisms and moderators that affect the relationship between teacher leadership and PA in adolescents, identifying self-determined motivation as one of the most relevant factors influencing this relationship. Recently, a study carried out with Spanish adolescents [26] indicated that perceptions of transformational teaching were positively associated with the most self-determined or autonomous forms of motivation (i.e., intrinsic motivation and identified regulation), and negatively associated with the less self-determined or controlled forms of motivation (i.e., external regulation and amotivation). In the specific PE context, evidence has consistently shown that self-determined motivation is related to the most positive consequences (i.e., cognitive, affective, and behavioural responses) while participating in classes (e.g., [39,49]). Moreover, self-determined motivation has been found to be the best determinant of the intention to be physically active outside PE (e.g., [50,51,52] and a robust predictor of PA levels at both younger and older ages [53,54].

In the school context, the passion students may feel toward an activity (e.g., PE classes) has been linked to their persistence on the activity [55,56], well-being, and positive affect [57]. In fact, the way students experience passion results in different experiences and outcomes. The dualistic model of passion proposed by Vallerand et al. [58] defines passion as a strong inclination toward a self-defining activity that one likes, finds important, and dedicates a significant amount of time and energy to doing. These authors proposed two types of passion: harmonious passion, where the activity remains under the person’s control, occupying an important but not overwhelming space; and obsessive passion, where the person is not in control of the activity and experiences an uncontrollable urge to engage in it. Results reveal that motivational climate drives the types of passion [57]. In fact, Vallerand and colleagues [58,59,60,61,62] argued that the social environment is instrumental in the development of different types of passion, which in turn may lead to differential outcomes. For example, Bonneville-Roussy et al. [63] found that students who perceived their teachers as autonomy supportive (a task-oriented climate) displayed higher levels of harmonious passion and persistence in their domain; in contrast, when students perceived their teacher as controlling (an ego-oriented climate) they were more prone to developing an obsessive passion that could lead them to drop out of the activity. Accordingly, an ego climate may facilitate obsessive passion, whereas a task climate is likely to lead to the development of harmonious passion (e.g., [64,65]). In addition, harmonious passion has been related to higher levels of well-being and more self-determined motivation in students, whereas obsessive passion has been linked to lower levels of life satisfaction and general well-being (for a review, see [66]). In the Spanish school context, PE teachers’ harmonious passion was positively associated with their transformational teaching style and, in turn, negatively related to experiencing burnout [67]. 

Based on previous theoretical and empirical findings on factors related to participation in school-based PE and leisure-time PA, and in order to improve our knowledge about the consequences of students’ perceptions of having a transformational PE teacher, the aim of the present study was to examine whether a transformational teaching practice predicts students’ future intentions to continue to be physically active outside of the school environment, as well as their levels of leisure time PA, through perceptions of motivational learning climate, passion, and self-determined motivation. 

**Figure 1:**
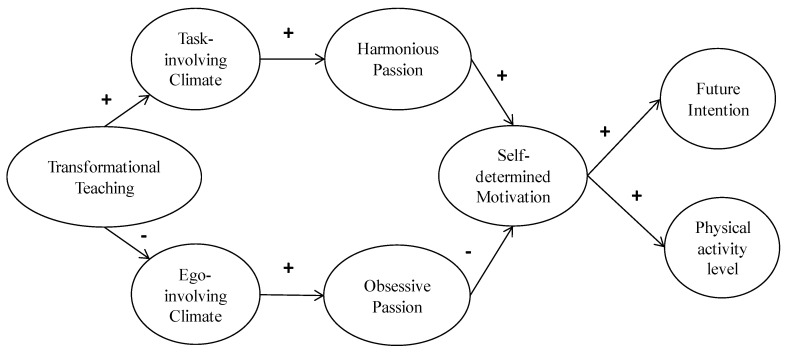
The hypothesized model of the interrelationships between PE students’ perceptions of the social environment, passion, self-determined motivation, future intention, and physical activity level.

In particular, we hypothesized that: (1) students’ perceptions of a transformational teaching style will be positively associated with a task-involving learning climate, whereas being a transformational teacher will be negatively associated with students’ perceptions of an ego-involving learning climate; (2) perceptions of a task-involving learning climate created by the teacher will predict harmonious passion; in contrast, perceptions of an ego-involving learning climate will be positively related to obsessive passion. In turn, (3) harmonious passion will be positively associated with self-determined motivation towards PE classes, whereas obsessive passion will be negatively related to it. Finally, (4) self-determined motivation will be positively related to future intentions to continue to be physically active and to the reported PA level in leisure time (see Figure 1).

## 2. Materials and Methods 

### 2.1. Design and Participants

This research had a non-experimental quantitative, correlational and cross-sectional design. 

A total of 2210 high school students (1145 males, 1065 females) ranging in age from 16 to 20 years (mean (M) = 17.42; standard deviation (SD) = 0.77), were recruited in 2015 from 40 secondary schools across the Valencian region (Spain) to participate in this study. Compulsory secondary education in Spain (aged between 12–18 years) consists of four years, and if a student wants to attend university, a further two years of Baccalaureate (Spanish *Bachillerato* is equivalent to 11th and 12th Grade in the US, and Year 12 and 13 in the UK) is required. PE classes of Grade 11 (*n* = 1830, 867 males, 963 females; M_age_ = 17.26, SD = 0.70) and Grade 12 (*n* = 380, 198 males, 182 females M_age_ = 18.18, SD = 0.62) were targeted for inclusion in this study. It is mandatory in Spain for all Grade 11 students to take part in PE and it is an optional subject for all Grade 12 students. In the present study, the number of students ranged from 24 to 30 per class. Considering previous research [68], municipalities, in which schools were located, were classified as urban areas if ≥20,000 residents, and rural areas if <20,000 residents. There were 29 schools from urban areas and 11 from rural areas.

Practice was assessed for 65 PE teachers (41 males) ranging in age from 30 to 63 years (M = 41.45; SD = 8.35). The years of experience as PE teachers ranged from 5 to 35 (M = 15.12; SD = 8.30).

### 2.2. Measures

Demographic information. Students provided data relating to their gender, age, and grade.

Transformational teaching. Students’ perceptions of their teachers’ behaviours associated with teaching style were measured using the Spanish version [26] of the Transformational Teaching Questionnaire (TTQ) [27]. The TTQ begins with the stem “My physical education teacher...” and uses a 5-point rating scale with anchors 1 (Not at all), 2 (Once in a while), 3 (Sometimes), 4 (Fairly often), and 5 (Frequently). The TTQ includes four subscales (each one made up of 4 items) to measure individualized consideration (e.g., “Shows that s/he cares about me”), idealized influence (e.g., “Acts as a person that I look up to”), intellectual stimulation (e.g., “Creates lessons that really encourage me to think”), and inspirational motivation (e.g., “Is enthusiastic about what I am capable of achieving”). The higher the self-reported value in TTQ the more the transformational teaching style is perceived. Previous research conducted in the physical domain has supported the internal reliability and predictive validity of this measure (e.g., [26,27]).

Learning climate. To assess students’ perceptions of the motivational climate created by the PE teacher, the participants completed selected items of the Spanish version [69] of the Perceived Motivational Climate in Sport Questionnaire-2 (PMCSQ-2) [70], which was adapted for use in PE classes for this study. Items assessed students’ perceptions of the degree to which the motivational climate of their respective classes is characterized in terms of two higher-order dimensions (task- and ego-involving climate). The participants responded to the nine items that ask them to rate what the environment is like in their PE classes in general, on a 5-point Likert scale ranging from 1 (strongly disagree) to 5 (strongly agree). The stem for each question was: “In my PE class…” An example of a task-involving dimension item is “The teacher encourages students to help each other”. An example of an item reflecting an ego-involving dimension is “Only the top students ‘get noticed’ by the teacher”. Previous studies have confirmed adequate internal reliability and factorial validity for this scale (e.g., [70,71]).

Passion. PE students’ passion was measured using a Spanish version [67] of the Passion Scale [58] with the items adapted to the PE domain for the present study. The scale comprises 16 items of which six measure harmonious passion (e.g., “Physical education allows me to live memorable experiences”), six measure obsessive passion (e.g., “I have almost an obsessive feeling for being in physical education classes”), and four criteria items that reflect the definition of passion. Specifically, participants are asked to report the extent to which they value their PE classes, devote time to PE classes, love PE classes, and view PE classes as a passion. PE students responded to all items on a 5-point Likert scale from 1 (strongly disagree) to 5 (strongly disagree). The Passion Scale has shown high levels of validity and reliability [58,67,72].

Self-determined motivation. Students’ self-determined motivation was assessed using two subscales of the Spanish version [73] of the Perceived Locus of Causality Scale (PLOC) [74], in which students responded to 8 items (divided into 2 subscales made up of 4 items each) that measure intrinsic motivation and identified regulation. The scale was headed by the phrase “I participate in this physical education class…” and the responses are rated on a 5-point Likert scale ranging from 1 (totally disagree) to 5 (totally agree). Exemplar items include “Because PE is fun” (intrinsic motivation) and “Because I want to learn sport skills” (identified regulation). Previous research has confirmed the reliability of the instrument both with Spanish [73] and English samples [74]. Aligned with SDT [46] and past research (e.g., [75]) in this study intrinsic motivation and identified regulation dimensions were combined to represent self-determined motivation.

Future intentions for PA. Intentions to continue being physically active in the future were assessed with the three adapted items from the work of Chatzisarantis et al. [76] adapted to the physical activity and/or sports for this study. Specifically, the participants responded to three items (“I am determined to continue doing physical activity and/or sports the next few months”, “I intend to do physical activity and/or sports the next few months”, and “I plan to do physical activity and/or sports the next few months”). Responses were indicated on a 5-point Likert scale ranging from 1 (very unlikely) to 5 (very likely). Previous work has suggested acceptable internal reliability of this scale in the case of young adolescents in the PE context [75,76,77].

Leisure-time PA level. Weekly PA was evaluated using the Spanish version of the Global Physical Activity Questionnaire survey (GPAQ) [78]. This survey allows researchers to assess moderate to vigorous-intensity physical activities performed during leisure time through a weekly estimation of the total energy expenditure (MET • minutes/week). One metabolic equivalent (MET) is the energy expenditure of sitting quietly and is approximately equivalent to 3.5 mL of oxygen per kilogram of body weight per minute [79]. The GPAQ questionnaire has been satisfactorily used among Spanish adolescents in previous research (e.g., [10]).

### 2.3. Procedure

Before collecting the data, permission to conduct the study was obtained from the authors’ ethical committee of the University of Valencia (Spain). The ethical approval code of the University of Valencia Ethical Committee is: 1259844.

The Spanish Ministry of Education, Research, Culture and Sports provided us with a list of high schools in the Valencian region. Schools were randomly selected (simple random sampling). A letter was sent to the head of each school, informing them about the goals of the investigation and requesting their collaboration. The majority of the schools contacted expressed interest in participating in the investigation (95.2% agreement). Students were provided with verbal information about the investigation and gave their informed consent before data collection. Consent was also sought from the PE teacher to conduct the research with their students. The parental consent was not obtained as at the time of the study it was not mandatory for participants aged 16 or over years of age in Spain. There was no form of professional development for teachers prior to the evaluation and they continued with their normal teaching style. The data were collected two months after the PE lessons were started to ensure that the students’ perceptions of the transformational teaching and motivational climate had been established. The students were requested to respond voluntarily, and anonymously to a package of questionnaires before their scheduled PE lesson, taking approximately 20 min to complete it. The surveys were administered by at least one investigator who encouraged students to answer honestly and ask any questions they had. The participants’ response rate to the survey was about 90%. PE teachers were not present during the survey administration.

### 2.4. Statistical Analyses

Descriptive statistics, Cronbach alpha coefficients and Pearson correlations were analysed using IBM SPSS Statistics version 20 (IBM Corp., Armonk, NY, USA). To examine whether the correlations between the hypothesized model variables were similar in both genders, a hypothesis contrast test was performed using the Fisher *r* to *z* transformation. The percentage of missing data was very small (<0.05%). To analyse the hypothesized model, we followed the two-step approach recommended by Anderson and Gerbing [80]. First, confirmatory factor analyses with LISREL 8.80 (Scientific Software International, Inc., Lincolnwood, IL, USA) [81] were performed with the hypothesized measurement model to determine whether the indicators were related to the latent factors satisfactorily. Second, after a satisfactory fit was achieved for the measurement model, we tested the fit of the structural model. The data were analysed using the robust maximum likelihood analysis as this procedure offers more accurate standard errors when the data are not normally distributed, and is also sensitive to missing data considerations. The polychoric correlation and the asymptotic covariance matrices were used as the input for the analyses. Multiple fit indices including the chi-square index (χ^2^), non-normed fit index (NNFI), comparative fit index (CFI), standardized root mean square residual (SRMR) and root mean square error of approximation (RMSEA) were employed to assess the adequacy of the model. Values close to or greater than 0.95 for the NNFI and CFI, and values of (or less) than 0.08 and 0.06 for the SRMR and RMSEA (and its 90% confidence interval) respectively, indicated a good fitting model to the data [82]. 

With the study sample size, we expected to have enough statistical power to detect relevant relationships between the variables of the model. We estimated that assuming a small effect size (f^2^ = 0.02) for a maximum number of 45 predictors, and a probability of Type I error of 0.05 to obtain a statistical power of 0.90, a sample size of 1809 individuals would be required [83].

## 3. Results

### 3.1. Descriptive Statistics, Scale Reliabilities and Bivariate Correlations

Descriptive statistics, internal consistency and correlations between the study’s variables appear in Table 1. The participants exhibited moderate average scores on all the scales (except for obsessive passion), above the response scale’s nominal midpoint. All the scales showed acceptable alpha coefficients (from 0.74 to 0.96). Transformational teaching was moderately correlated (*p* < 0.01) with a task-involving climate, both types of passion, self-determined motivation, and future intentions to being active. Transformational teaching also shared a low but significant positive association (*p* < 0.01) with ego-involving climate and PA level. Except for a not significant relation with PA level (*p* > 0.05), perceptions of a task-involving climate shared the same, but a generally weaker pattern of significant associations as transformational teaching style. The ego-involving climate was marginally and positively associated (*p* < 0.01) with harmonious and obsessive passion, self-determined motivation, future PA intentions and PA level. Both types of passion were positively related (*p* < 0.01) to self-determined motivation, future PA intentions and PA level. Finally, self-determined motivation was positively related to future PA intentions and PA level (*p* < 0.01) (see Table 1).

The results of the gender differences in the correlations showed that there were no significant differences (z < 1.96) in the majority of the studied variables (see Table 2), so the hypothesized model analyses were carried out with the overall sample.

### 3.2. The Measurement and Structural Models

**Figure 2:**
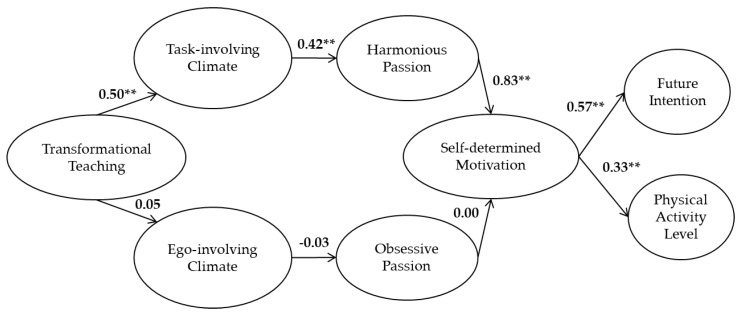
The structural model of the interrelationships between PE students’ perceptions of the social environment, passion, self-determined motivation, future intention and physical activity level. All coefficients are standardized. Factor indicators are not represented for reasons of simplicity of presentation. The correlation between the error of the indicators of harmonious and obsessive passions (r = 0.61) are not shown. ** *p* < 0.01.

A full measurement model was tested for divergent validity of the latent factors and an adequate fit was obtained: χ^2^ (1099) = 5458.35, *p* = 0.001, NNFI = 0.985; CFI = 0.986; SRMR = 0.051; RMSEA = 0.043 (90% CI = 0.042; 0.044). The fit indices for the hypothesized structural model was acceptable, χ^2^ (1118) = 5627.55, *p* = 0.001, NNFI = 0.985; CFI = 0.986; SRMR = 0.057; RMSEA = 0.044 (90% CI = 0.042; 0.045). The standardized parameter estimates are shown in Figure 2. The results revealed partial support for the hypothesized model. Perceptions of transformational teaching were positively and moderately related with task-involving climate and not significantly related to ego-involving climate. In line with the proposed model, there was a significant and positive path between task climate and harmonious passion, which in turn was a significant and strong predictor of self-determined motivation. The association between ego climate and obsessive passion was not significant as well as the association between this type of passion and self-determined motivation. Finally, self-determined motivation was a positive predictor of the future intention of being active and the level of PA outside of school hours (see Figure 2).

The standardized indirect effects indicated that transformational teaching positively predicted harmonious passion (β = 0.21, *p* < 0.01) through task-involving climate. Transformational teaching also positively predicted self-determined motivation (β = 0.17, *p* < 0.01) through task climate and harmonious passion; and positively predicted future intention to be active (β = 0.10, *p* < 0.01) and PA level (β = 0.06, *p* < 0.01) through task climate, harmonious passion and self-determined motivation. Moreover, the perception of a task-involving climate was positively linked to self-determined motivation (β = 0.35, *p* < 0.01) through harmonious passion. Task climate also positively predicted future intention for being active (β = 0.20, *p* < 0.01) and PA level (β = 0.12, *p* < 0.01) through harmonious passion and self-determined motivation. Finally, harmonious passion positively predicted future intention for being active (β = 0.47, *p* < 0.01) and PA level (β = 0.28, *p* < 0.01) through self-determined motivation.

## 4. Discussion

Grounded in a transformational teaching framework, as a conceptual extension of transformational leadership theory [29], AGT [32], and SDT [46], this study examined how PE teachers’ transformational leadership style can predict students’ PA participation. To this end, we examined the associations between PE students’ perceptions of teachers’ transformational teaching style and two PA outcomes (i.e., PA participation in their leisure time and intention to engage in PA in the future), testing the mediational role of motivational learning climates (i.e., task- and ego-involving climate), types of passion (i.e., harmonious and obsessive), and self-determined motivation in this relationship. Overall, the results of this study provided partial support for the hypothesized model confirming most of the proposed relationships in terms of regression paths involving a task-involving climate, harmonious passion, and self-determined motivation. By contrast, the regression paths from transformational teaching to an ego-involving learning climate, from an ego-involving climate to obsessive passion, and from obsessive passion to self-determined (or autonomous) motivation for learning were not confirmed.

As expected, and in accordance with our first hypothesis, the study results showed a positive relationship between a transformational teaching style and a task-involving learning climate. During engagement in PE activity, students who perceive that their PE teacher inspires them to take action to create a shared vision that is aligned with the goals and needs of the group, and students who feel that the PE teacher understands their strengths and needs and encourages them to use multiple perspectives to deal with new and old issues were also more likely to perceive a task-involving climate created by the PE teacher. These results offer support for the framework proposed by Duda and Balaguer [43] integrating leadership behaviours and motivational climate, and they extend previous findings in the sport (e.g., [44,84]) and school contexts (e.g., [45,85]) by confirming that individual perceptions of a transformational leadership style were associated with a positive and adaptive motivational climate. 

Additionally, and consistent with our second and third hypotheses and the research by Bonneville-Roussy et al. [63], students’ perceptions of a task-involving learning climate were positively related to harmonious passion, which in turn was associated with self-determined motivation. That is, students who perceive that their PE teacher promotes learning and cooperation in their PE classes, proposes challenging tasks, and emphasizes positive evaluations for personal improvement are more likely to have their PE passion under control and operate in harmony with the other school-tasks, thus behaving with a full sense of volition and choice (self-determination).

Consistent with previous studies in the sport context (e.g., [65,86]), our results showed that an ego-involving learning climate was unrelated to being obsessively passionate in PE classes and they also rule out the possibility that this type of passion may be related to lower self-determined student motivation. Instead, our findings confirmed that a task-involving learning climate is more conducive to developing students’ harmonious passion, leading to the achievement of positive and desirable outcomes. SDT theorists (e.g., [87]) suggest that, although both harmoniously passionate and self-determined students enjoy the activity, they differ in that harmonious passion is also embedded in the individual’ self-concept because of its roots in autonomous internalization [58]. With this in mind, a PE student can be self-determined to participate in PE classes without being passionate about them. In this regard, findings show that students who are harmoniously passionate are more likely to experience choice and self-determination and engage in PE classes because they are genuinely interested in the activity. 

Our results support the distinction between the two types of passion proposed by Vallerand et al. [58] because obsessive passion was not found to be significantly associated with self-determined student motivation. The results also support the hypothesis that harmonious passion would arise when a task-involving learning climate is created, which would subsequently be related to positive outcomes. Finally, and consistent with our expectations, students who were self-determined for learning reported higher levels of PA in their leisure time, as well as a greater intention to continue to be physically active, confirming our fourth hypothesis.

This research provides evidence of the importance of adopting a transformational teaching style as a correlate of a task-involving learning climate that encourages students to become harmoniously passionate. It might also be related to important outcomes for students, such as the promotion of PA behaviours outside school, the intention to remain active and, ultimately, their adoption of an active lifestyle. Our findings also extend previous literature [26] by exploring the mechanism through which transformational teaching may be associated with self-determined motivation for learning in PE students. Specifically, transformational teaching positively predicted self-determined motivation through task climate and harmonious passion. Furthermore, transformational teaching also positively predicted the future intention to be active and the PA level through task climate, harmonious passion, and self-determined motivation.

The positive findings support the importance of PE teachers in the acquisition of an active lifestyle beyond PE classes, and they reinforce the proposal by Sallis and McKenzie [14], who suggested that students’ experiences in PE classes would affect the intention to continue to be physically active in the future and even, as our findings show, the level of PA participation in their leisure time. Moreover, the present findings support the idea that health-based PE should be understood in a complete way, not only as a context that seeks to promote high levels of PA, but also as a way to encourage active lifestyles through personally relevant and enjoyable activities that positively affect students’ motivation to perform them in their leisure time [88]. In this regard, being a transformational PE teacher and creating a positive classroom climate could be important factors in improving students’ harmonious passion and self-determination to participate in PE class (i.e., feeling that the activity is fun), further facilitating students’ intention to continue the activity and improving their PA participation outside school hours.

Relevant research found that teachers who display transformational teaching behaviours improve positive classroom climate, which in turn promotes learning effectiveness [89]. Along these lines, the results of this study corroborate that PE teachers’ transformational teaching style, creating a positive learning climate (i.e., task-involving climate), could be a requirement for successful teaching that can enhance harmonious passion and self-determined motivation and promote high levels of PA participation outside PE classes (i.e., in their leisure time). Moreover, the results could serve as a reference for PE teachers to enhance positive experiences in PE classes, so that the benefits of being physically active could transcend the school environment.

## 5. Strengths and Limitations

Strengths of this study include the use of reliable and validated instruments. Furthermore, we propose, in the school context, an integrated model of how teachers’ transformational teaching style can be related to students’ PA participation in their leisure time, using three important theoretical frameworks at the same time: transformational leadership theory [29], achievement goal theory [32], and self-determination theory [46]. Another important strength of this study is the high participation rate (about 90%). We consider that allowing students to complete questionnaires at a convenient time and place for them can result in higher participation rates.

On the other hand, some limitations of this study include the fact that the information is obtained through self-reported measures and that the study design is cross-sectional. In addition, students may over-report their PA participation outside school and their intention to continue with the PA practice because of social desirability. The timing of the data collection (before the PE class) may even have affected the response in terms of the future intention to continue PA and sports. With this in mind, it is important for future research to adopt a longitudinal approach to identify the corresponding causal relationships and include the use of some objective measures, such as accelerometers that allow us to assess the level of PA after school hours, or observational measures of the teacher’s leadership style. Furthermore, this study did not include any assessments by the PE teachers, and so we do not know whether the teacher perceives that he or she is using transformational teaching behaviours in the PE classes. We only rely exclusively on the students’ perceptions. Finally, another limitation of this study is that we did not analyse the pedagogical strategies of the PE teachers or other aspects of the lessons, such as the type of content the teachers were covering. These limitations lead us to interpret the results with caution.

## 6. Practical Implications

This research offers empirical evidence of what Ames suggested when she pointed out that, “… changing classroom structures may also require changing teachers’ goals for children’s learning, belief systems, or broader views about school learning” [31]. From an applied perspective, we emphasize the need for teacher training programs (e.g., [31,90]) that include psychosocial issues and, more specifically, transformational teaching behaviours (e.g., emphasizing that teachers should act as role models, by highlighting the importance of teachers showing consistency between what they say and what they do, and the values they promote in their classes. For example, if the teacher asks his or her students to be on time, the teacher should be on time. If teachers ask their students to respect their peers, teachers should be respectful of their students, erasing the role of privileges associated with the traditional view of the teacher’s role), as well as creating task-involving climates to help teachers be more effective during PE instruction (e.g., integrating TARGET structures into lesson planning, for example, by stimulating teachers to provide real choices where decisions are based on effort, and not on ability evaluations). 

The intervention strategy would follow the guidelines established by Beauchamp et al. [90] adopting an approach from a social learning perspective [91]. Thus, the intervention program should clarify what it means to be a transformational teacher, providing an overview of the concepts of the transformational leadership style and its components (i.e., idealized influence, inspirational motivation, intellectual stimulation, and individualized consideration); it should identify the strategies used by transformational teachers in their daily interactions with students; and, finally, it should offer feedback on the strategies these teachers use in their current work contexts. Furthermore, the intervention program should be designed to teach teachers to create a task-involving learning climate, according to the TARGET structures, encouraging them to set more mastery goals, use a variety of tasks, and focus more on effort and self-referenced improvement. 

A better understanding of the consequences of transformational teaching and a task-involving climate in PE may help to improve children’s and adolescents’ PA participation. The PE teacher plays a crucial role in the development of positive/negative experiences in PA practice that can be crucial in promoting PA engagement outside of school, and official institutions have the responsibility of helping them by providing specific tools and skills to carry out their mission.

## 7. Conclusions

This study confirms that PE teachers should apply transformational leadership in the teaching process to promote PA participation in their students’ leisure time and the intention to continue to be physically active. Furthermore, and as proposed in the literature reviewed [e.g., [45,85], being a transformational teacher is related to good motivational learning climates (task-involving climate), extending our knowledge about the importance of adopting a task-involving climate as a positive and appropriate motivational climate to further promote harmonious passion, which is related to self-determined motivation. Additionally, enhancing students’ self-determined motivation could increase PA participation and even the intention to remain physically active in the future. In sum, using a transformational teaching style could affect the degree of participation in PA in their leisure time and the intention to engage in these activities in the future, by creating a task-involving motivational climate that reinforces the student’s harmonious passion, which is associated with self-determined motivation. 

## Figures and Tables

**Table 1:** Descriptive statistics, internal consistency and correlations between study variables.

Variables	Mean	SD	α	1	2	3	4	5	6	7
1.Transformational teaching	3.60	0.91	0.96	-						
2. Task-involving climate	3.61	0.66	0.78	0.43 **	-					
3. Ego-involving climate	3.93	0.79	0.74	0.08 **	0.05 **	-				
4. Harmonious passion	3.31	0.84	0.87	0.49 **	0.44 **	0.13 **	-			
5. Obsessive passion	2.36	0.86	0.81	0.31 **	0.18 **	0.06 **	0.54 **	-		
6. Self-determined motivation	3.71	0.80	0.91	0.50 **	0.40 **	0.11 **	0.73 **	0.45 **	-	
7. Future PA intentions	3.89	1.04	0.95	0.22 **	0.16 **	0.09 **	0.44 **	0.28 **	0.50 **	-
8. PA level (METs • min/week)	2350.46	2352.49	-	0.08 **	0.03	0.08 **	0.25 **	0.26 **	0.25 **	0.49 **

Note. PA = Physical activity. Range 1–5 except for metabolic equivalents (METs) that are 0–19200. α = Cronbach’s alpha reliability coefficient. ** *p* < 0.01.

**Table 2:** Results of values of correlation differences across gender for the study variables.

Correlation Variables	Males Correlation	Females Correlation	z
Transformational Teaching—Task-involving climate	0.46 **	0.42 **	1.16
Transformational Teaching—Ego-involving climate	0.15 **	0.01	3.11 **
Task-involving climate—Harmonious passion	0.48 **	0.42 **	1.82
Ego-involving climate—Obsessive passion	0.09 **	0.02	1.66
Harmonious passion—Self-determined motivation	0.69 **	0.75 **	−3.07 **
Obsessive passion—Self-determined motivation	0.43 **	0.44 **	−0.37
Self-determined motivation—PA level	0.22 **	0.23 **	−0.31
Self-determined motivation—Future PA intention	0.48 **	0.50 **	−0.73

Note. z = standardized test statistic, ** *p* < 0.01.
